# Different sets of *TaCKX* genes affect yield-related traits in wheat plants grown in a controlled environment and in field conditions

**DOI:** 10.1186/s12870-020-02713-9

**Published:** 2020-10-29

**Authors:** Karolina Szala, Hanna Ogonowska, Boguslawa Lugowska, Barbara Zmijewska, Renata Wyszynska, Marta Dmochowska-Boguta, Waclaw Orczyk, Anna Nadolska-Orczyk

**Affiliations:** 1 Department of Functional Genomics, Plant Breeding and Acclimatization Institute – National Research Institute, Radzikow, 05-870 Blonie, Poland; 2 Danko Hodowla Roslin Ltd., Choryn 27, 64-005 Koscian, Poland; 3 Plant Breeding Strzelce Ltd., Co. - IHAR Group, Konczewice 1, 87-140 Chelmza, Poland; 4 International Institute of Molecular and Cell Biology, Trojdena 4, 02-109 Warsaw, Poland; 5 Department of Genetic Engineering, Plant Breeding and Acclimatization Institute – National Research Institute, Radzikow, 05-870 Blonie, Poland

**Keywords:** *TaCKX*, *TaNAC*, Wheat, Field, Laboratory conditions, Cytokinin, Phytohormone crosstalk, Grain yield

## Abstract

**Background:**

*TaCKX* wheat gene family members (GFMs) encode the enzyme cytokinin oxidase/dehydrogenase (CKX), which irreversibly degrades cytokinins. The genes are important regulators of cytokinin content and take part in growth and development, with a major impact on yield-related traits. The goal of this research was to test whether these genes might be differentially expressed in the field compared to laboratory conditions and consequently differently affect plant development and yield.

**Results:**

We compared expression and crosstalk of the *TaCKX* GFMs and *TaNAC2-5A* gene in modern varieties grown in a growth chamber (GC) and in the field and looked for differences in their impact on yield-related traits. The *TaNAC2-5A* gene was included in the research since it was expected to play an important role in co-regulation of these genes. The range of relative expression levels of *TaCKX* GFMs and *TaNAC2-5A* gene among tested cultivars was from 5 for *TaCKX8* to more than 100 for *TaCKX9* in the GC and from 6 for *TaCKX8* to 275 for *TaCKX10* in the field. The range was similar for four of them in the GC, but was much higher for seven others and *TaNAC2-5A* in the field. The *TaCKX* GFMs and *TaNAC2-5A* form co-expression groups, which differ depending on growth conditions. Consequently, the genes also differently regulate yield-related traits in the GC and in the field. *TaNAC2-5A* took part in negative regulation of tiller number and CKX activity in seedling roots only in controlled GC conditions. Grain number and grain yield were negatively regulated by *TaCKX10* in the GC but positively by *TaCKX8* and others in the field. Some of the genes, which were expressed in seedling roots, negatively influenced tiller number and positively regulated seedling root weight, CKX activity in the spikes, thousand grain weight (TGW) as well as formation of semi-empty spikes.

**Conclusions:**

We have documented that: 1) natural variation in expression levels of tested genes in both environments is very high, indicating the possibility of selection of beneficial genotypes for breeding purposes, 2) to create a model of an ideotype for breeding, we need to take into consideration the natural environment.

**Supplementary Information:**

The online version contains supplementary material available at 10.1186/s12870-020-02713-9.

## Background

Wheat is one of the most economically important cereal crops [1]. It can be cultivated in a wide range of environmental conditions and is rich in nutrition components. It provides approximately 20% of protein in the human diet [2]. The large, hexaploid genome, which is composed of three A, B and D diploid genomes, is a rich reservoir of genes determining yield-related traits [3]. However, the increase of wheat yield has remained moderate or even stagnate within the last two decades [4, 5]. This cereal species is much less studied compared to rice and maize [6].

*CKX* GFMs encode the enzyme cytokinin oxidase/dehydrogenase (CKX), which irreversibly degrades cytokinins [7], and therefore strongly regulates cytokinin content in different organs of plants. Since cytokinins play a diverse role in plant development and affect a number of agriculturally important processes [8, 9], the *CKX* genes influence yield-related traits. In cereals it was documented by means of silencing of selected *CKX* in rice [10], in barley [11, 12] and in wheat [13] leading to an increased level of cytokinins, affecting yield components. The phytohormone also regulates changes in gene expression of cytokinin-induced genes to mediate its pleiotropic effect [9, 14].

The number of *CKX* GFMs varies depending on species. In bread wheat 11 to 14 gene family members have been proposed [15–18]. *TaCKX2* underwent gene duplication [15] and the resultant genes were assigned as *TaCKX2.1* and *TaCKX2.2* based on the Ensembl Plants database [19] and phylogenetic analysis by Ogonowska et al. [16]. The numbering of *TaCKX* GFMs was recently revised taking advantage of the most up-to-date databases (including IWGSC RefSeq v2.0), their homology with closely related monocot species including barley and diploid genome specificity [20]. Based on these data, there are 13 *TaCKX* GFMs basically numbered as *TaCKX1*, *TaCKX2.1*, *TaCKX2.2.1*, *TaCKX2.2.2*, *TaCKX2.2.3*, *TaCKX3* (*6*), *TaCKX4*, *TaCKX5*, *TaCKX7*, *TaCKX8* (*11*), *TaCKX9* (*10*), *TaCKX10* (*9*) and *TaCKX11* (*3*), and 12 of them are allocated to each of the three chromosomes associated with the A, B and D genomes, giving the number of 35 homoeologous genes. The exception is *TaCKX2.2.2* allocated only to D genome. This new numbering of the *TaCKX* genes is applied in this publication and the former numbers are shown in brackets.

The *CKXs* exhibit distinct patterns of organ- and development-specific expression. In our earlier research on *HvCKX* in barley we found that expression patterns of the genes indicate their role in growth and reproductive development [21]. Therefore, the first step of our investigation of the *CKX* genes in wheat was focused on expression specificity in different organs and different developmental stages [16]. Based on these results the genes were classified to four groups: i) leaf-specific *TaCKX9*, *TaCKX5*, *TaCKX4*; ii) specific to inflorescence and developing spike *TaCKX1* and *TaCKX2*; iii) seedling root-specific *TaCKX10*, *TaCKX7* and iv) expressed at various levels in all tested organs *TaCKX11*, *TaCKX3*, *TaCKX8* [16]. The *TaCKX* GFMs co-operated inside and among organs. The effect of *TaCKX1* silencing was further investigated in 7 DAP (days after pollination) spikes [13]. Various levels of *TaCKX1* silencing in T_1_ and T_2_ generations influenced different modes of co-expression with other *TaCKX* GFMs and parameters of yield-related traits. Only lines with strongly silenced *TaCKX1* and associated with this strong down-regulated *TaCKX11* and up-regulated *TaCKX2.1* and *TaCKX9* were characterized as high yielding. The content of most of the cytokinins in spikes 7 DAP of silenced T_2_ lines significantly increased, and their interaction with other phytohormones was demonstrated. Each of the tested yield-related traits was regulated by various up- or down-regulated *TaCKX* GFMs and phytohormones.

Cytokinins regulate gene transcription in target organs and developmental stages through a wide range of transcription factors (TF) [22]. One of the largest groups of plant TFs involved in cytokinin-dependent regulation is the family of NAC (for NAM, ATAF, and CUC) TFs. It has been documented that NACs are involved in the regulation of important agronomic traits [23–26]. Overexpression of nitrate inducible wheat *TaNAC2-5A* increased root growth, the rate of nitrate uptake under hydroponic conditions and in the field increased grain yield, grain nitrogen (N) concentration, and N harvest index under various N supply levels [23]. This gene was included in our research as an important regulator of cytokinin activity.

Experiments in controlled environments, in contrast to field conditions expose plants to constant irradiance during the day, constant temperature and artificial soil conditions. The growth parameters and the morphology of Arabidopsis plants, grown under artificial light, independent of the source, were significantly different compared with plants grown in natural conditions [27]. Distinct epistatic interactions on flowering time in barley were observed in field and in semi-controlled conditions [28]. Moreover, plant hormones differently regulated grain development in normal and under abiotic stress conditions [29–32]. It was also reported that yield stability genes are differentially expressed in the field compared to laboratory conditions [33].

Transgenic solutions to increase yield in wheat seemed to be very promising, but no transgenic cultivar has yet been approved [5]. On the other hand, natural variation is proposed as a profitable rich source of research objects to understand the responses mediated by jasmonate signalling [34]. We have been testing two alternative strategies of changing *TaCKX*-dependent cytokinin regulation in order to increase grain yield in wheat. One is based on the modifications of the *TaCKX* genes’ expression via RNAi-based gene silencing [13]. The second one, presented here, explores the possibility of selection of natural variants differing in expression of *TaCKX* GFMs in wheat genotypes/breeding material. Differences revealed in co-regulation of yield-related traits by these GFMs in different environments, could lead to selection of appropriate genotypes in breeding programmes dedicated to specific environment. Therefore in the first part of the study we examined the range of expression of the *TaCKX* GFMs in 7 DAP spikes and in seedling roots among cultivars and breeding lines in a growth chamber and in the field. We analysed co-expression among themselves and with *TaNAC2-5A* and their influence on yield-related traits in both environments. In the next part of this research we will investigate how expression patterns of selected marker genes are inherited and whether they are consistent with inheritance of yield-related traits.

## Results

### Expression of *TaNAC2-5A* in different organs of developing wheat plants

**Fig. 1:**
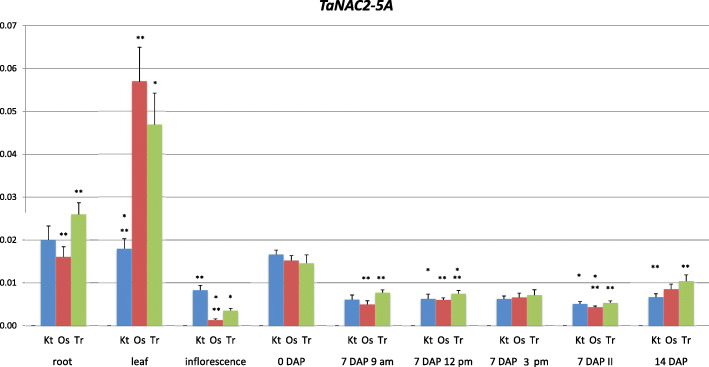
Specificity of expression of *TaNAC2-5A* gene in developing organs of wheat. ** – significant at *p* ≤ 0.01; * – significant at *p* ≤ 0.05; II – second spike

The level of relative expression of *TaNAC2-5A* (*NAC2*) was highest in leaves, seedling roots and 0 DAP spikes, ranging from 0.015 up to 0.057 (Fig. 1). This wide range was mainly dependent on the large differences of expression of the gene among three tested cultivars. For two of them, the cultivars Ostka and Trappe, the expression levels of the *NAC2* in leaves was about three times higher than in Kontesa. Interestingly, opposite data of expression levels in the same cultivars were found in inflorescence, where they were several times lower.

There were no significant differences in expression of *NAC2* in 0 DAP spikes among cultivars. Expression levels of *NAC2* in 7 DAP and 14 DAP spikes ranged from 0.005 to 0.01. There were some differences between cultivars, but not between the first and the second spike or daily times of sample collection.

### Range of variability of expression of *TaCKX* GFMs and *NAC2* in growth chamber and the field

**Fig. 2:**
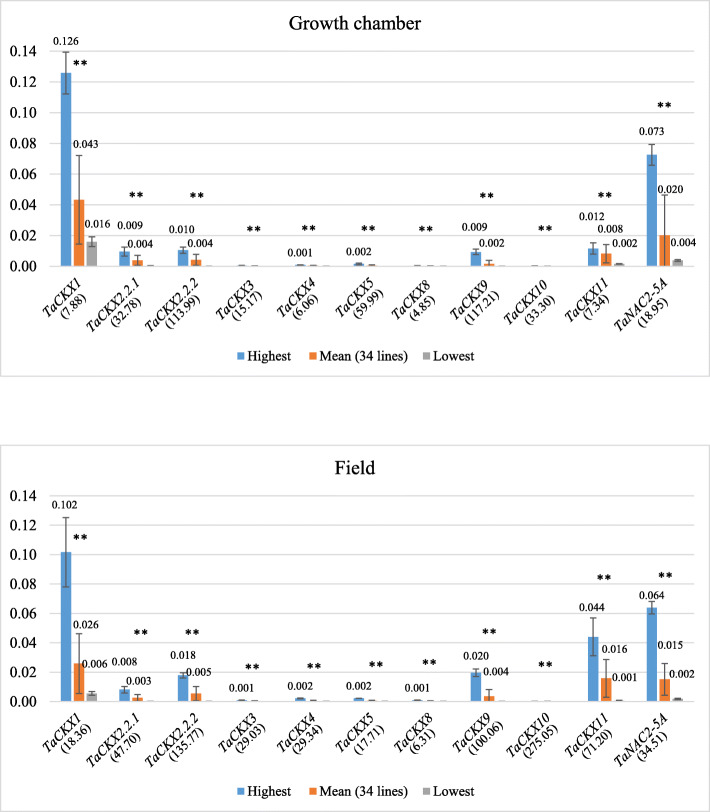
Highest, mean and lowest data of expression levels of *TaCKX* GFMs and *NAC2* in 7 DAP spikes of 34 breeding lines growing in growth chamber and in the field. ** – significant at p ≤ 0.01 (between highest and lowest). (…) – quotient of variability

Expression levels of *TaCKX* GFMs and *NAC2* were measured in 7 DAP spikes of 34 breeding lines growing in a growth chamber (GC) and in the field. Highest, mean and lowest values of relative expression are presented in Fig. 2. The level of the *CKX1* mean relative expression was higher in 7 DAP spikes from the GC (0.043) compared to the same material from the field (0.026). However the highest (0.126) and the lowest (0.016) values obtained for the same gene in the GC indicated a 7.88-fold difference (quotient of variability), while the values (0.102 and 0.006) in the field showed 18.36-fold difference.

Similar to *CKX1*, mean expression of *CKX2.2.1* and *NAC2* (*TaNAC2-5A*) were higher in the GC (0.0038 and 0.020) compared to the field (0.0025 and 0.015). However for most of the *TaCKX* GFMs (*CKX2.2.2*, *CKX3*, *CKX5*, *CKX8, CKX9*, *CKX10*, *CKX11*) the mean values of expression were higher in the field compared to the GC. The range of expression variability between breeding lines in 7 DAP spikes growing in both conditions was similar for *CKX2.2.1*, *CKX2.2.2*, *CKX5* and *CKX9*, and showed big differences for *CKX1*, *CKX3*, *CKX4*, *CKX5*, *CKX10*, *CKX11* and *NAC2* in the field conditions. The highest variability between expression levels of the genes from the GC compared to the field ranging from 5 to 10 times was observed for *CKX4*, *CKX10* and *CKX11*. *CKX1* was highly expressed followed by *CKX11*, *CKX2.2.2* and *CKX9* in both growing conditions. The mean level of *NAC2* expression was similar to *CKX11* in the field (0.016 and 0.015 respectively) and two times higher in the GC (0.020 and 0.008 respectively). Standard deviation of gene expression levels among three biological replicates were higher in spikes from the field compared to the GC.

### Co-expression of *TaCKX* GFMs in growth chamber and the field

**Fig. 3:**
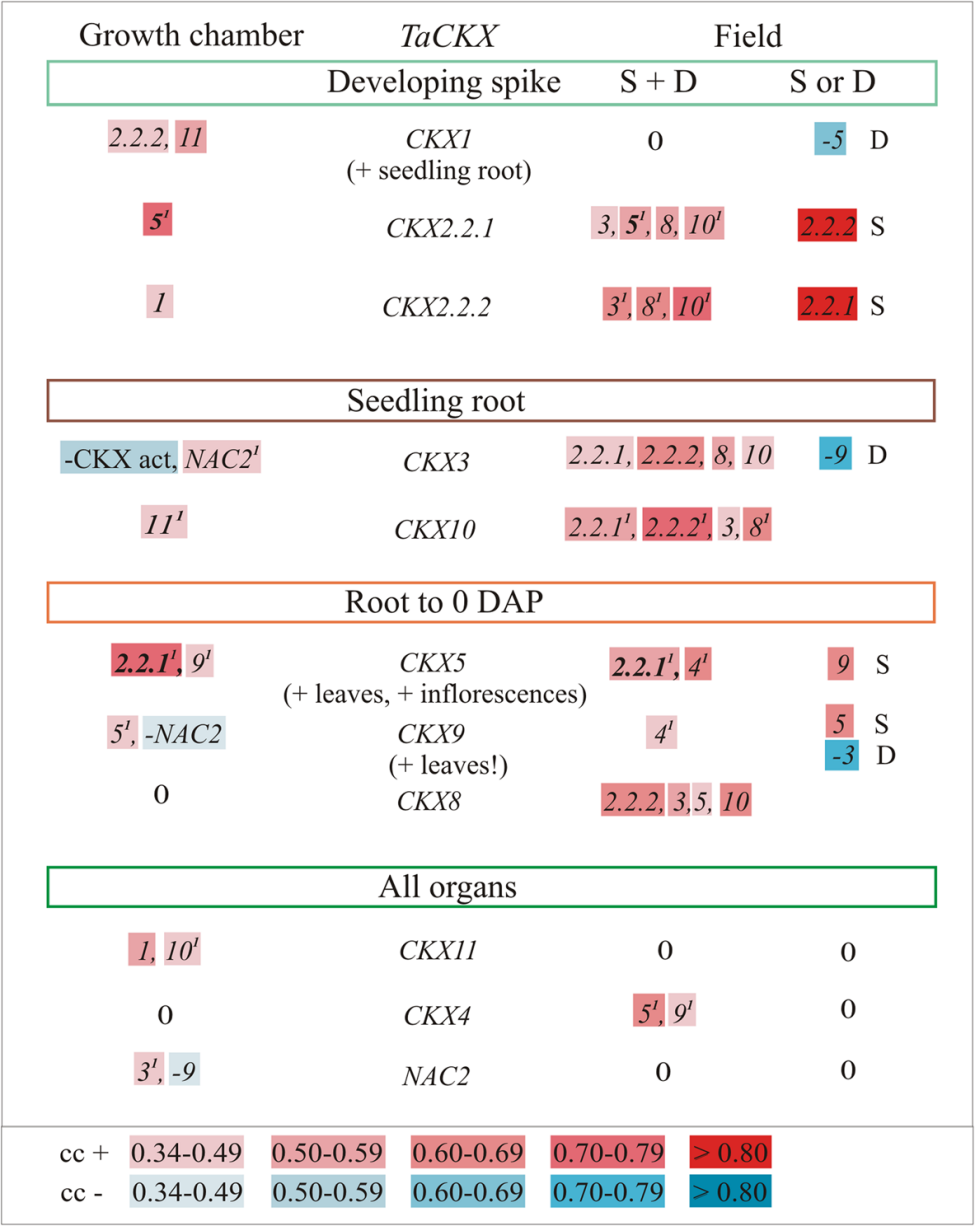
Co-expression of *TaCKX* GFMs and *NAC2* in 7 DAP spike from the growth chamber and from the fields of Strzelce (S) and Danko (D) together (S + D) or separately (S or D) based on correlation coefficient (cc). All cc are significant at *p* ≤ 0.05. Gradation of positive cc in red and negative cc in blue; “0” – lack of correlation; ^1^– non-parametric; bold – cc in the growth chamber and in the field

Correlation between expression levels of various *TaCKX* GFMs was different in both growth conditions, GC and the field (Fig. 3, Table S2). Since breeding lines were grown on two different fields of two Plant Breeding Companies Strzelce (S) and Danko (D), correlations are presented for both conditions together (S + D) and separately (S or D). Generally, in field conditions the groups of mutually correlated genes were larger than in GC conditions and co-expression of a few of them was specific to the field (S or D). According to expression specificity, the *TaCKX* GFMs were divided into four groups. The first one contained the genes specifically expressed in developing spikes; the genes from the second group were expressed in seedling roots; the third were specific in younger plant organs from seedling roots to 0 DAP spikes and the fourth to all organs tested.

Expression level of spike specific *CKX1* significantly correlated with expression levels of *CKX2.2.2* and *CKX11* in the GC, but no correlation with others was found in spikes from both fields. Otherwise *CKX5* showed specific negative co-expression with *CKX1*, when spikes were grown on D field. *CKX2.2.2*, whose expression correlated with *CKX1* in the GC, was strongly correlated with *CKX3, CKX10* and *CKX8* in both fields, and individually with *CKX2.2.1* in S field. *CKX2.2.1* was co-expressed with *CKX5* in both growing conditions. The gene was also co-expressed with *CKX3*, *CKX8* and *CKX10* in both fields and individually with *CKX2.2.2* in S field. Expression of root-specific *CKX3* from the second group of genes positively correlated with expression of *NAC2* and negatively with CKX activity in the GC and with *CKX2.2.1*, *CKX2.2.2*, *CKX8* and *CKX10* in the fields as well as strongly negatively with *CKX9* in D field. Another root-specific, *CKX10*, exclusively correlated with *CKX11* in the GC and with *CKX2.2.1*, *CKX2.2.2*, and *CKX8* in the fields as in the case of *CKX3*. Moreover, expression level of both, *CKX3* and *CKX10* showed a mutual correlation in the fields conditions. The *CKX5*, which was strongly expressed in younger organs, especially in seedling roots, leaves and inflorescences, co-expressed with *CKX2.2.1* in both conditions, with *CKX9* in GC and with *CKX4* in the fields as well as with *CKX9* in S field. Expression of the next one from this group, *CKX9,* which was especially high in leaves, was positively correlated with *CKX5* and negatively with *NAC2* in GC but positively with *CKX4* in both fields, and positively with *CKX5* in S field but negatively with *CKX3* in D field. No correlation was found between expression of *CKX8* and any of the tested genes in GC. However, the *CKX8* was positively correlated with *CKX2.2.2*, *CKX3, CKX5* and *CKX10* in both fields conditions.

The last group represented genes expressed in all organs. Interestingly, expression of *CKX11* correlated with expression of *CKX1* and *CKX10* in GC. There was no correlation of this gene with others in the field conditions. Conversely, the level of *CKX4* expression positively correlated with *CKX5* and *CKX9* in both fields, but there was no correlation in GC. There was a positive correlation between expression of *NAC2* and *CKX3* and a negative correlation with *CKX9* in GC. No correlation of *NAC2* with others was found in the field.

### Expression of *TaCKX* GFMs and *NAC2* and CKX activity in 7 DAP spikes differently affect yield-related traits in both growth conditions

**Fig. 4:**
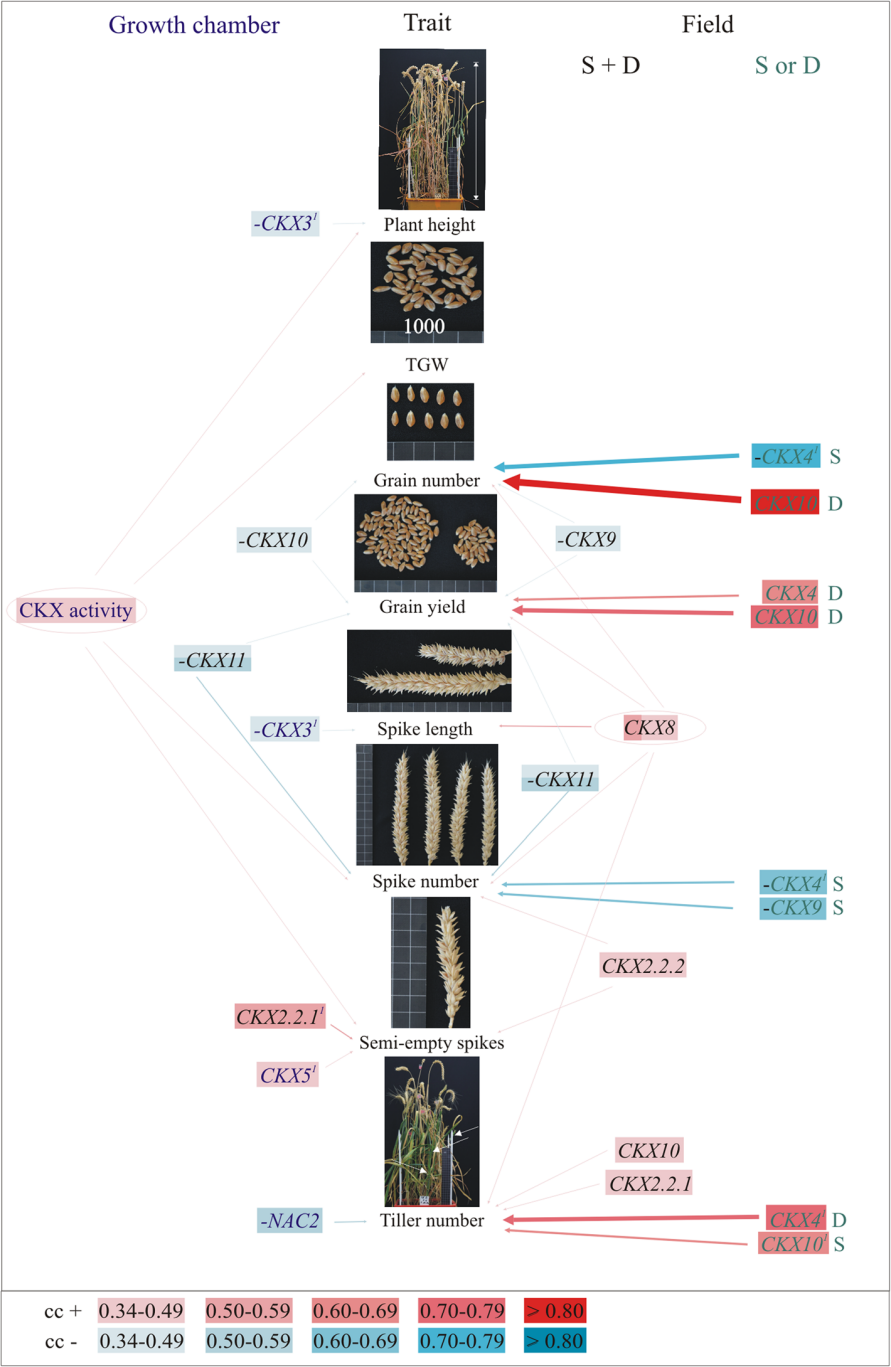
Interconnections between *TaCKX* GFMs and *NAC2* expression, and CKX activity in 7 DAP spikes from the growth chamber (left) and the fields (right) with yield-related traits based on significant cc. The cc from the fields of Strzelce (S) and Danko (D) counted together (S + D) or separately (S or D). Gradation of positive cc in red and negative cc in blue; ^1^– non-parametric analysis; “-“negative correlation

In the GC, plant height and TGW were positively correlated with CKX activity in 7 DAP spikes (Fig. 4, Table S3). However, the plant height and the spike length were negatively correlated with *CKX3*. Enzyme activity was also positively correlated with number of spikes and number of semi-empty spikes. Moreover, the number of semi-empty spikes is positively correlated with expression of *CKX2.2.1* and *CKX5,* and tiller number is negatively correlated with *NAC2*. Grain number and grain yield in the same growth conditions are negatively correlated with *CKX10*. Otherwise, in the fields conditions both grain number and grain yield are negatively correlated with expression of *CKX9,* and positively correlated with *CKX8*, but the grain yield is negatively correlated with *CKX11* (spikes from both fields). Additionally grain number is strongly negatively correlated with *CKX4* expression in spikes from S field and very strongly positively correlated with *CKX10* in case of spikes from D field. Otherwise grain yield is positively regulated by *CKX4* and *CKX10* in case of D field. The *CKX8* expressed in spikes from both fields is not only positively correlated with grain number and grain yield but also with spike length, spike number and tiller number. Both spike number and number of semi-empty spikes are positively correlated with *CKX2.2.2* measured in spikes collected from both fields. Individually, spike number is negatively correlated with *CKX4* and *CKX9* only in case of spikes from S field and tiller number is positively correlated with *CKX4* and *CKX10* measured in spikes from D and S fields respectively.

### Expression of *TaCKX* GFMs and *NAC2* and activity of CKX in seedling roots affect yield-related traits

**Fig. 5:**
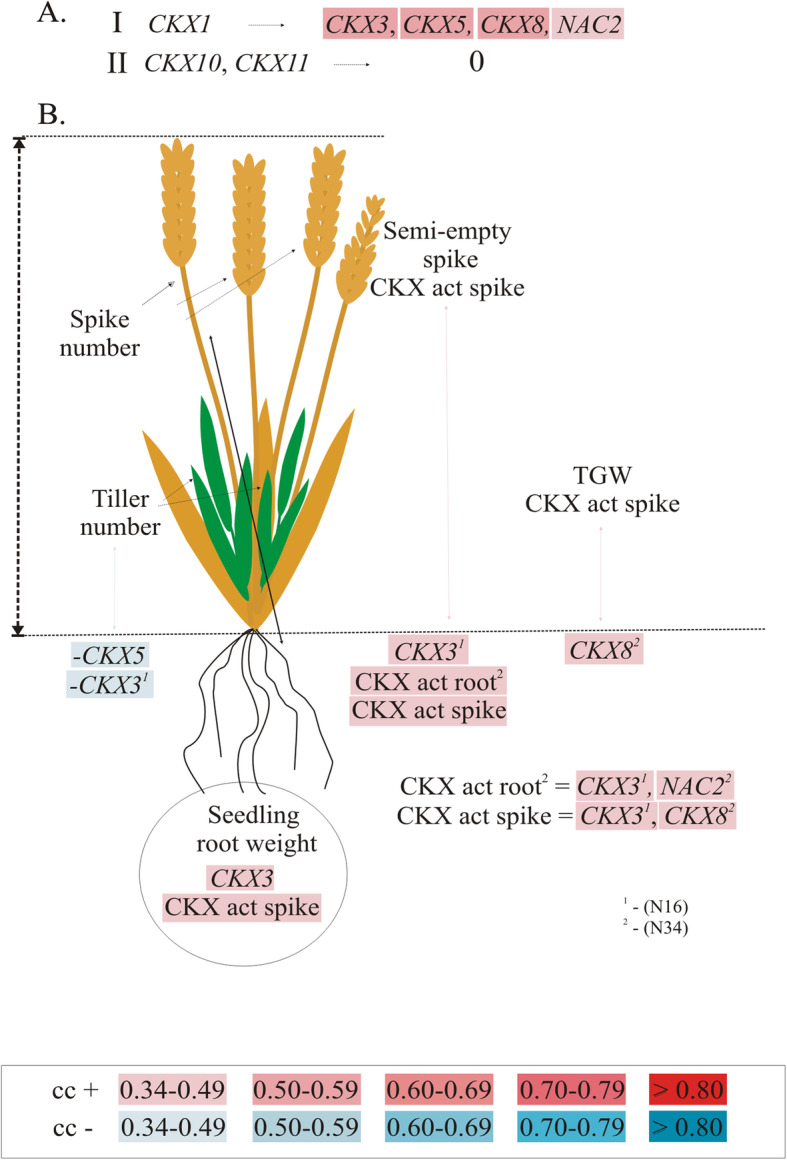
Influence of *TaCKX* GFMs and *NAC2* expression, and CKX activity (CKX act) in seedling roots on seedling root weight and yield-related traits. Coexpression of *TaCKX* GFM and *NAC2* in seedling roots (**a**) and their cooperation with above ground yield-related traits (**b**) based on significant cc (p ≤ 0.05). 0 – no correlation; N16, N34 – cc for 16 and 34 breeding lines respectively; gradation of positive cc in red and negative cc in blue

Expression of *CKX1* in seedling roots was positively and strongly correlated with *CKX3, CKX5*, *CKX8* and *NAC2* (Fig. 5a, Table S4). There was no correlation between *CKX10* and *CKX11* and other genes expressed in this organ.

*CKX3* expressed in seedling roots is the most frequently interacting gene with yield-related traits in the GC (Fig. 5b, Table S4). Its expression positively correlated with seedling root weight and semi-empty spikes, but negatively with tiller number. Positive correlation of the *CKX3* is accompanied by CKX activity in spikes, and in the case of semi-empty spikes, additionally with CKX activity in roots. Moreover, *CKX3* and *CKX8* are positive regulators of spike CKX activity and, with *NAC2*, positively correlated with CKX activity measured in seedling roots. Expression of *CKX8* is positively correlated with CKX activity in spikes and TGW. Expression of *CKX5* is negatively correlated with tiller number.

## Discussion

### *TaNAC2-5A* is expressed in different organs of wheat plants

*TaNAC2-5A* (*NAC2*) was included into this research on regulation of yield-related traits of wheat by the *TaCKX* gene family as a possible important regulator of these genes and the corresponding processes. This nitrate-inducible *NAC2* encoding gene was reported by He et al. [23] to be an important regulator of root growth and wheat yield. To relate *NAC2* expression to our previous research on expression specificity of *TaCKX*, we performed expression experiments similar to those we did with *TaCKX* [16]. Relative expression levels of *NAC2* in roots, leaves and 14 DAP spikes reported by He et al. [23] were comparable with our results. The *NAC2* is also expressed in organs newly tested by us: inflorescence, 0 DAP and 7 DAP spikes, but at a level lower than for 14 DAP spikes. Generally, the gene is expressed in all organs tested. We also demonstrated that, especially in the case of leaves, inflorescence and root expression, levels of *NAC2* differed considerably between cultivars.

### Large differences in *TaCKX* GFMs and *NAC2* expression levels among breeding lines allow them to be selected for breeding purposes

The differences between the highest and the lowest expression levels of tested *TaCKX* GFMs and *NAC2* in breeding lines were significant and ranged from several to over a hundred times. These differences were much bigger for six of the genes expressed in 7 DAP spikes growing in the field compared to GC. This is consistent with the observation that stress inducible genes were expressed more strongly in field than in laboratory conditions [33]. These differences in expression might be the result of allelic variation among the tested genes, their genetic background and cross-talk with environments. Allelic variation among *TaCKX* family genes corresponding with yield-related traits was reported by others [35–37]. The data indicate the possibility of using this parameter for selection in the breeding process.

### Selected *TaCKX* GFMs are differentially expressed in field and GC conditions

The similar range of expression variability among breeding lines for *CKX2.2.1*, *CKX2.2.2*, *CKX8* and *CKX9* in the GC and in the field might indicate that expression levels of these genes are independent of growth conditions. The data indicating stable expression being maintained by these genes in changing conditions are in agreement with newly published results of *TaCKX1* silencing in the case of both *CKX2.2* [13]. There was strongly decreased expression of *TaCKX1* gene in silent lines while expression of *CKX2.2* remained at the same level. Otherwise, the impact of unstable field growth conditions on expression of *CKX1*, *CKX3*, *CKX4*, *CKX5*, *CKX10*, *CKX11* and *NAC2* was significant. Indeed, *TaCKX1* silencing caused strong down-regulation of expression of *CKX11* and up-regulation of *CKX5* [13]. It may be dependent on the same feedback mechanism between induction of expression of selected *CKX* genes by cytokinin and their function [38]. It was already documented that constant irradiance during the day in controlled environments and lack of light-day transitions may significantly influence plant phenotype [27]. Consequently, many group of genes determining yield in normal and stress conditions, genes encoding chloroplast located proteins or shade avoidance (including genes involved in hormonal regulation, light and flowering) are differentially expressed in growth chamber versus field conditions [33, 39]. Expression of *CKX* as well as biosynthetic *IPT* genes might also be regulated by the levels of available macronutrients, such as nitrate and phosphate [40], and biotic/abiotic stress conditions in the field.

### *TaCKX* GFMs and *NAC2* in field or controlled environments form different co-expression groups

The *TaCKX* GFMs and *NAC2* are predominantly co-expressed in the GC and in the field in different groups. Only two of them co-work with each other in both conditions: *CKX2.2.1* expressed in developing spikes and *CKX5* highly expressed in seedling roots, inflorescences and leaves [16]. This cooperation may indicate their importance in regulation of cytokinin level between roots and spikes during plant growth and development independently of growth conditions.

What is remarkable, *CKX1* highly expressed in developing spikes and roots, and *CKX11* and *NAC2* expressed in all organs formed co-expression groups with other genes only in GC conditions. It does not mean that the genes are not susceptible to different nutrition conditions since *NAC2* was shown to be inducible by nitrate [23]. However, it is possible that expression of these genes might be independent of main environmental factors as the source of light and light-day transitions as well as changing temperatures and soil moisture. *CKX11* highly expressed in all organs, co-worked with *CKX1* and *CKX10* in the GC conditions but not in the field, although their expression in the field was seven times higher. This strong co-regulation of *CKX11* with *CKX1* was already observed in our research with silencing the *TaCKX1* performed in GC conditions [13]. Conversely *CKX4* expressed in all organs and *CKX8* expressed in younger organs, which did not show any co-expression with others in GC, were co-regulated by others in the field. Therefore, these genes might take part in regulation of cytokinin content by involving others in more stressful conditions.

Significantly larger were groups of *TaCKX* genes co-expressed in the unstable, field conditions. *CKX8* expressed in younger organs was the most frequently co-working with others, mainly those expressed in developing spikes and roots. However, the gene co-works with *CKX5* belonging to the same expression group, which might link coregulation of expression of other genes in both growth conditions. Expression of *CKX2.2.1* and *2.2.2* specifically expressed in developing spikes is correlated with the same root-specific *CKX3* and *CKX10* as well as *CKX8* specific to younger organs but only in field conditions. Both *CKX2.2* genes correlate with different genes in GC, *CKX2.2.1* with *CKX5* and *CKX2.2.2* with *CKX1*, which might indicate their different detailed functions (discussed below).

Both root-specific *CKX3* and *CKX10* are co-regulated with both, spike-specific *CKX2.2* and *CKX8* specific to all organs, and each other in the field, but in GC these genes specifically co-work with GC-specific *NAC2* and *CKX11* respectively.

### Yield-related traits are regulated by different *TaCKX* GFMs and *NAC2*, depending on the natural or controlled environments

As in the case of co-expression, more *CKX* genes were involved in regulation of yield-related traits, especially grain number, grain yield, spike number and tiller number in the field conditions than in GC. The most active in the field was *CKX8* expressed in all organs co-regulating with *CKX9* grain number, with *CKX9* and *CKX11* grain yield, with *CKX11* spike number, with *CKX2.2.1* and *CKX10* tiller number and as a single spike length. In most cases these traits are positively regulated by *CKX8* but negatively by co-expressed *TaCKX* genes, which indicates their predominant role in maintaining hormonal homeostasis. Interestingly some traits like grain number, grain yield, spike number and tiller number are strongly regulated by *CKX4* and *CKX10* but this regulation is field dependent. Presumably both genes are strongly dependent on environmental conditions since both research fields differ in type of soil and weather conditions.

The same traits, grain number and grain yield, are regulated in GC negatively by *CKX10*. None of the genes regulates the same yield-related trait in both growth conditions. Similarly as in the study by Jablonski et al. [13], grain yield and spike number in GC were negatively regulated in 7 DAP spikes by *CKX11* which, in cooperation with *CKX2.1*, influenced down-regulation of free base, active *trans*-zeatin (tZ) and *N*^6^-(Δ2-isopentenyl) adenine (iP) but up-regulation of benzyladenine (BA). In the same research, spike number in GC-grown, control plants was regulated by *CKX2.2.1* as well, which together with *CKX1* and *CKX5* up-regulated tZ and iP and down-regulated BA. Some of our data do not appear comparable, because in Jablonski et al. [13] only one cultivar with a restricted number of *TaCKX* genes and yield-related traits was tested. However, it was shown that the genes form different co-expression groups in 7 DAP spikes of *TaCKX1* silent and not-silent plants whereby they control the content of various cytokinins and determine final yield-related traits. Different coregulation of both *CKX2.2* genes in the GC and the field resulted in their various impact on yield-related traits. *CKX2.2.1* together with *CKX5* is involved in formation of semi-empty spikes in GC but *CKX2.2.2* alone has such involvement in the field. *CKX2.2.1* together with *CKX8* and *CKX10* positively regulates tiller number in the field as well. None exhibits the same role in the GC and in the field. Zhang et al. [35] reported that *TaCKX6*, renamed by [20] as *TaCKX2.2.1-3D*, is an orthologue of *OsCKX2* regulating rice yield [10]. The cultivar, which has an 18-bp deletion, showed decreased expression of this gene, which was associated with a greater TGW than in other haplotypes. There is a small incompatibility in maximum transcript level, which was reached at 8 DAP in the study by Zhang et al. [35] and was increasing from 0 DAP through 7 DAP and reaching the maximum at 14 DAP in our research (later stages were not tested). This increasing expression in developing spikes up to 14 DAP better explains the possible role of the gene in grain filling, which is an essential factor of greater TGW. Besides this, we did not find any correlation of expression of this gene with TGW, although its expression together with *CKX5* positively influenced formation of semi-empty spikes in GC and with two others tiller number in the field. Therefore the joint effect of *TaCKX* family genes on regulation of yield-related traits depending on growth conditions is important. However, expression of *TaCKX2.2.1-3A* (originally *TaCKX2.4*) reduced by RNAi [41] showed a strong correlation with grain number in T_3_ due to an increased grain number per spike. Similar result is obtained in our research, in which *CKX2.2.1* together with *CKX5* positively regulates formation of semi-empty spikes in GC, although the same trait was regulated by *CKX2.2.2* in the field. Moreover, function of *TaCKX2.2.1-3A* is closer to their orthologue *OsCKX2* in rice, where is also associated with grain number [10]. According to Li et al. [41], different functions of *TaCKX2.2.1-3D* and *TaCKX2.2.1-3A* genes are the result of different locations on chromosomes 3D and 3A respectively. We suppose that these differences might be additionally modulated by possible individual co-operation with other genes within an individual genetic background.

There were two more characterized *TaCKX* family genes, both demonstrated as determinant of yield-related traits. Isolated by Lu et al. [36], the novel allele *TaCKX6a* allocated to the *TaCKX2.1* gene family [16] and renamed as *TaCKX2.1-3D* [20] showed, as in our earlier research [13], significant correlations with grain size, weight and grain filling rate in recombinant inbred lines. In the second report one out of two alleles of *TaCKX4* found by Chang et al. [37] was positively associated with chlorophyll content and grain yield. The authors did not show any expression data. According to our research, the gene is expressed in all tested organs of developing wheat plant in the GC, showing higher expression in leaves, but their expression was low compared to others [16]. We did not find any correlation of their expression with yield-related traits in the CG. However we found higher expression of this gene in field conditions comparing to the GC as well as their strong correlations with yield-related traits, which was dependent on the individual field conditions.

### Selected *TaCKX* GFMs and *NAC2* expressed in seedling roots regulate some yield-related traits

As already reported [16], *CKX1* expressed in seedling roots forms a co-expression group with *CKX3*, *CKX5*, *CKX8* and *NAC2* in the same organ. Moreover, the *TaCKX* GFMs and *NAC2* gene expressed in seedling roots formed different co-expression groups with that expressed in developing spikes. It means that the genes also participate in coordinated development of the above ground part of the plant, which is dependent on growth conditions. Such cooperation of *TaCKX* GFMs is obvious, since they regulate CKX enzyme activity, which inactivates cytokinins, plant hormones known to act opposingly in shoot and root growth [42]. An example is *CKX3*, highly expressed in seedling roots, which is positively correlated with seedling root weight but negatively correlated with plant height and spike length. *NAC2*, controlling with *CKX3*, CKX activity in roots, when expressed in spikes, negatively correlated with tiller number. Moreover, *CKX3* with *CKX5* expressed in roots negatively regulated tiller number, but *CKX3* with CKX activity in roots positively determined formation of semi-empty spikes, and *CKX8* expressed in roots positively influenced TGW. Such cooperation of seedling root expressing genes with those expressed in organs from the aerial part of wheat plants and their influence on yield traits was also documented in our earlier research [13, 16].

Generally, overexpression of *CKX* in roots of other plant species leads to enhanced root growth by reduction of cytokinin levels [43–46]. By contrast, an elevated cytokinin level inhibits root growth but promotes shoot growth in rice [47]. Among the *CKX* GFMs in rice, Gao et al. [48] indicated a key role of *OsCKX4* in the initiation of crown roots by interaction between auxin and cytokinin biosynthesis. Mao et al. [49] underlined the role of NAC transcription factors on rice root development. Overexpressed *OsNAC2* increased expression of several *IPT*, decreased *OsCKX4* and *OsCKX5* and increased cytokinin level. Therefore *OsNAC2* stimulated cytokinin accumulation by promotion of cytokinin biosynthesis and repression of *CKX* expression. This was affected by the binding of *OsNAC2* to the promoter of *OsCKX4*. In our research, wheat *TaCKX4* orthologous to *OsCKX4* was not found to correlate with seedling root weight, possibly because of its very low level of expression in roots as well as other organs of wheat plants. However, we showed strong correlations between the *CKX4* gene expression and yield-related traits, which was dependent on field conditions. The second orthologue of rice, *TaCKX5*, together with *CKX3*, both expressed in roots, was negatively correlated with tiller number, but the same trait was negatively influenced by *NAC2* expressed in spike.

## Conclusions

It was previously indicated that yield stability genes are differentially expressed in the field compared to growth chambers [33]. These differences might mean that many genetically modified plants carrying single, agronomically important genes cannot be applied in practice [5, 50]. Therefore, to indicate which *TaCKX* family genes might be applicable in breeding programmes, it is important to characterize them in both environments, laboratory conditions and in the field. Indeed, we found large differences in the levels of expression, crosstalk of those genes and correlations with agronomic traits in modern varieties and breeding lines dependent on the environmental conditions. We have documented that to create a model of an ideotype for breeding, we need to take into consideration the field environment. Natural variation in expression levels of tested genes, especially in the field, was very high, indicating the possibility of selection of beneficial genotypes/phenotypes for breeding purposes. Such selection based on variable expression of selected genes, resulting from mutations in the tested genetic background and cross-talk with others, can be carried out among recent breeding material in their field environments. We suppose that combining beneficial genotypes selected in such environments could help breeders to exploit available genetic variation and optimize further yield potential in their regional soil and climate conditions. Therefore, the next step of our research is to show if and how expression of *TaCKX* GFMs is inherited in subsequent generations.

## Methods

### Plant material

The experimental plant material was collected from 34 breeding lines and cultivars of common wheat (*Triticum aestivum* L.) delivered by two Plant Breeding Companies: Strzelce Ltd., Co. - IHAR-PIB Group (23 breeding lines) and Danko Hodowla Roslin Ltd. (11 breeding lines). Selected genotypes differed in productivity and other interesting breeding features, such as resistance to diseases, plant height, protein content (including gluten), and starch in grain or seed hardness. The plants were grown in two different growth conditions: in a growth chamber and in a field.

**Fig. 6:**
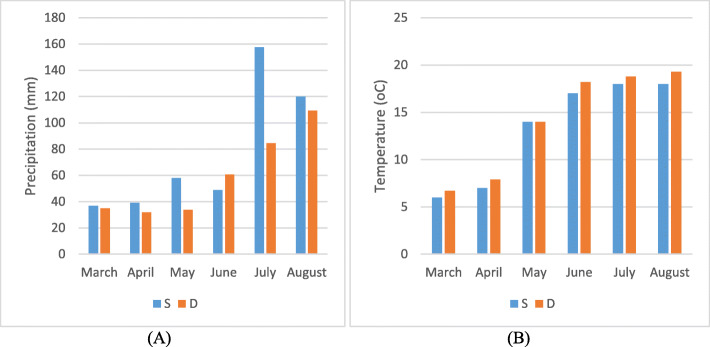
Weather conditions for particular months in two Plant Breeding Companies, Strzelce (S) and Danko (D): total precipitation (**a**); mean air temperature (**b**)

Field conditions: The field experiments were conducted on two experimental fields of Strzelce Company (Konczewice) and Danko Company (Choryn). The first is located in the north-central part of Poland (Kujawsko-Pomorski region) and the second in the west-central (Wielkopolska region), about 150 km from each other. Experimental plots were 5 m^2^ (= 5 × 1 m) and 400 grains were sowing per 1 m^2^. White beet and rape were the forecrops respectively. Nitrogen was applied twice at the beginning of spring vegetation and at the beginning of stem elongation in two doses (100–120 kg ha^− 1^ total). The soil was also fertilized by 20–50 kg ha^− 1^ phosphorus and 50–80 kg ha^− 1^ potassium, depending on the field requirements. Humidity and thermal conditions during the experiment are shown in Fig. 6. Water deficiency was supplemented when needed.

Germination of seeds and growth chamber conditions were performed as earlier reported [16]. The 5-days seedlings were replanted into pots with soil after earlier collection of seedling roots, which were cut 3 mm from embryo axis. The tissue samples: 5-day-old seedling roots and the middle parts of first spikes 7 days after pollination (7 DAP) were collected in three biological replicates from the growth chamber and from the field. In both environments spikes with the first 1–3 sticking out anthers from spikelets in the middle part were marked and collected after 7 days. Three biological replicates were selected randomly out of six in the growth chamber (one spike each) and out of whole plots in the field (three spikes each). These in the field were marked the same day and collected 7 days later. All these samples were started to collected at 9:00 am. Additional tissue samples used for testing *NAC2* expression were collected from three cultivars of common wheat: Kontesa, Ostka and Trappe according to Ogonowska et al. [16].

### RNA extraction and cDNA synthesis

Total RNA from 5-day-old seedlings’ roots and 7 DAP spikes was extracted using TRI Reagent (Sigma-Aldrich) according to the manufacturer’s protocol. Purification of RNA and cDNA synthesis was performed as described in Ogonowska et al. [16].

### Quantitative RT-qPCR

RT-qPCR analysis was performed for 11 target genes: *TaCKX1* (JN128583), *TaCKX2.2.1* (FJ648070)/*TaCKX2.2.2* (GU084177), *TaCKX3* (former *TaCKX6*) (JN128587), *TaCKX4* (JN128586), *TaCKX5* (Lei et al. 2008), *TaCKX8* (former *TaCKX11*) (JN128592), *TaCKX9* (former *TaCKX10*) (JN128591), *TaCKX10* (former *TaCKX9*) (JN128590), *TaCKX11* (former *TaCKX3*) (JN128585), *TaNAC2-5A* (AY625683). Sequences of primers designed for the genes tested are shown in Table S1. RT-qPCR reactions were assayed as described in Ogonowska et al. [16]. Each reaction was performed in three biological and three technical replicates. The two standard curves method with *ADP-ribosylation factor* (*Ref* 2*)* as a normalizer [51] was used for calculation of genes expression. Relative expression for each of the *TaCKX* family gene was calculated in relation to the control cultivar Ostka set as 1.00.

### Analysis of CKX activity

Measurements of CKX enzyme activity were performed in the same samples subjected to *TaCKX* gene expression analysis according to the procedure developed by Frebort et al. [52] and optimized for wheat tissues. The procedure is described in Zalewski et al. [12]. Concentration of the product was determined at the absorption spectrum ranged from 230 nm to 550 nm. The total protein concentration was estimated based on the bovine serum albumin standard curve according to the Bradford procedure [53].

### Measurement of yield-related traits

Morphometric measurement of yield-related traits of selected genotypes was performed. The described traits were: plant height, tiller number, spike number, semi-empty spike number, spike length, grain number, grain yield, TGW and seedling root weight.

### Statistical analysis

Statistical analysis was done using Statistica 13 (StatSoft) software. The normality of data distribution was tested using the Shapiro-Wilk test. The significance of changes was analysed using analysis of variance (ANOVA) and post-hoc tests. Correlation coefficients were determined using parametric correlation matrices (Pearson’s test) or a nonparametric correlation (Spearman’s test).

## Supplementary Information

**Additional file 1 Table S1**. Sequences of primers designed for amplification of the genes. **Table S2**. Correlations among expression of *TaCKX* GFMs and *NAC2* in 7 DAP spikes growing in growth chamber (A) and in the field (B) and in seedling roots (C). ^1^- non-parametric analysis; bold*– significant correlation at *p* ≤ 0.05. **Table S3**. Correlations among expression of *TaCKX* GFMs and *NAC2* in 7 DAP spikes from the growth chamber (A) and the field (B) on yield-related traits. ^1^- non-parametric analysis; bold* – significant correlation at *p* ≤ 0.05. **Table S4**. Correlations among expression of *TaCKX* GFMs and *NAC2* in seedling roots with seedling root weight and other yield-related traits with. ^1^- non-parametric analysis; bold* – significant correlation at p ≤ 0.05. **Table S5**. Correlations among expression of *TaCKX* GFMs and *NAC2* in 7 DAP spikes from the growth chamber (A) and the field (B) and expression in seedling root. ^1^- non-parametric analysis; bold* – significant correlation at p ≤ 0.05.

## Data Availability

All data generated or analysed during this study are included in this published article [and its supplementary information files].

## Abbreviations

*CKX1–11*: *TaCKX1 – TaCKX11*
*NAC2*: *TaNAC2-5A*
GFMs: Gene family members
GC: Growth chamber
CKX: Cytokinin oxidase/dehydrogenase
S: Plant Breeding Strzelce
D: Danko Hodowla Roslin
tZ: *Trans*-zeatin
iP: *N*^6^-(Δ2-isopentenyl) adenine
BA: Benzyladenine
TGW: Thousand grain weight
