# Supplementary material for: Different sets of TaCKX genes affect yield-related traits in wheat plants grown in a controlled environment and in field conditions
Source: BMC Plant Biol. 2020 Oct 29;20:496. doi: 10.1186/s12870-020-02713-9 (PMC7597040; doi:10.1186/s12870-020-02713-9)
Supplement: Supplementary file 1 — Additional file 1 Table S1. Sequences of primers designed for amplification of the genes. Table S2. Correlations among expression of TaCKX GFMs and NAC2 in 7 DAP spikes growing in growth chamber (A) and in the field (B) and in seedling roots (C). 1- non-parametric analysis; bold*– significant correlation at p ≤ 0.05. Table S3. Correlations among expression of TaCKX GFMs and NAC2 in 7 DAP spikes from the growth chamber (A) and the field (B) on yield-related traits. 1- non-parametric analysis; bold* – significant correlation at p ≤ 0.05. Table S4. Correlations among expression of TaCKX GFMs and NAC2 in seedling roots with seedling root weight and other yield-related traits with. 1- non-parametric analysis; bold* – significant correlation at p ≤ 0.05. Table S5. Correlations among expression of TaCKX GFMs and NAC2 in 7 DAP spikes from the growth chamber (A) and the field (B) and expression in seedling root. 1- non-parametric analysis; bold* – significant correlation at p ≤ 0.05. [file 12870_2020_2713_MOESM1_ESM.docx]

Table S1. Sequences of primers designed for amplification of the genes.

| *Ref 2* | Ta2291R | NCBI | GCTTCTGCCTGTCACATACGC | 165 |
| --- | --- | --- | --- | --- |
|  | Ta2291F |  | GCTCTCCAACAACATTGCCAAC |  |
| *TaCKX1* | TaCKX1_188R | | CCCAGGTACTCCTTGTACCCTAT | 188 |
|  | TaCKX1_188F | | GTCTACCCGCTCAACAAATCC |  |
| *TaCKX2.2.1* | TaCKX2_1_R_205 | | TATCACATACGCCATCCATGC | 205 |
|  | TaCKX2_1_F_205 | | TTGATCGCGGAGCTAATCCA |  |
| *TaCKX2.2.2* | TaCKX2_2_R_175 | | ATCGTATCCTGGCCTCCTCA | 175 |
|  | TaCKX2_2_F_175 | | TACCCCATGAACCGGAACAG |  |
| *TaCKX3* | TaCKX6_182R | | CCGTGCTTGAATGTCTGC | 182 |
|  | TaCKX6_182F | | CACAAAGGAGGAGAAGGAGATG |  |
| *TaCKX4* | TaCKX4_112_R | | CTCCAAAGTCACACCCTCTACAC | 112 |
|  | TaCKX4_112_F | | AAGAACACGCAGCATAGCAAC |  |
| *TaCKX5* | TaCKX5_3B_4R | | CATACATGACACCAACGTACATCTT | 150 |
|  | TaCKX5_3B_4F | | GTCCGATTTTTGAGAAGACTGATT |  |
| *TaCKX8* | TaCKX11_R_184 | | AGTCATGCACTGCAAACTCTATG | 184 |
|  | TaCKX11_F_184 | | GACCAAGAGCTTCTGATCTCAAT |  |
| *TaCKX9* | TaCKX10_R_167 | | ACATAAAGCAATTTACCTGGACTTG | 167 |
|  | TaCKX10_F_167 | | GAGCTAAGGGCTTGTGGGA |  |
| *TaCKX10* | TaCKX9_R_278 | | GTCCCTGTTCATGGGGTACA | 278 |
|  | TaCKX9_F_278 | | CCACGGTGGATCAGAAGCTC |  |
| *TaCKX11* | TaCKX3_150R | | GAATTAGAGTTCACGGCTTGATG | 150 |
|  | TaCKX3_150F | | TTGTCAAGGGACTGTAGTAGGG |  |
| *TaNAC2-5A* | TaNAC2_R | | GATGATGGAGCCCAAGGCGGAG | 100 |
|  | TaNAC2_F | | CTGGGTGCTCTGCCGGCTCTAC |  |

Table S2. Correlations among expression of *TaCKX* GFMs and *NAC2* in 7 DAP spikes growing in growth chamber (A) and in the field (B) and in seedling roots (C). ^1^- non-parametric analysis; bold*– significant correlation at p ≤ 0.05.

A

| N34. 7 DAP GC | *CKX1* | *CKX2.2.1 ^1^* | *CKX2.2.2* | *CKX3 ^1^* | *CKX4* | *CKX5^1^* | *CKX8* | *CKX9* | *CKX10 ^1^* | *CKX11* | *NAC2* |
| --- | --- | --- | --- | --- | --- | --- | --- | --- | --- | --- | --- |
| CKX act. | 0.16 | 0.14 | 0.04 | **-0.56*** | -0.17 | -0.11 | 0.08 | 0.24 | 0.13 | 0.22 | -0.19 |
| *CKX1* |  | 0.11 | **0.34*** | -0.35 | -0.17 | -0.11 | 0.03 | -0.20 | 0.25 | **0.59*** | 0.29 |
| *CKX2.2.1^1^* |  |  | 0.25 | -0.08 | -0.12 | **0.70*** | 0.10 | 0.13 | 0.03 | 0.20 | -0.13 |
| *CKX2.2.2* |  |  |  | 0.05 | 0.04 | 0.03 | -0.22 | 0.13 | 0.25 | 0.37 | 0.04 |
| *CKX3^1^* |  |  |  |  | 0.28 | -0.06 | 0.05 | -0.21 | -0.04 | -0.18 | **0.44*** |
| *CKX4* |  |  |  |  |  | -0.19 | 0.22 | 0.06 | 0.36 | 0.20 | 0.04 |
| *CKX5^1^* |  |  |  |  |  |  | -0.04 | **0.41*** | -0.06 | 0.13 | -0.18 |
| *CKX8* |  |  |  |  |  |  |  | 0.02 | 0.00 | -0.24 | 0.03 |
| *CKX9* |  |  |  |  |  |  |  |  | -0.19 | 0.11 | **-0.40*** |
| *CKX10^1^* |  |  |  |  |  |  |  |  |  | **0.41*** | 0.07 |
| *CKX11* |  |  |  |  |  |  |  |  |  |  | 0.34 |

B

| N34. 7 DAP field *^1^* | *CKX1 field* | *CKX2.2.1 field* | *CKX2.2.2 field* | *CKX3 field* | *CKX4 field* | *CKX5 field* | *CKX8 field* | *CKX9 field* | *CKX10 field* | *CKX11 field* | *NAC2 field* |
| --- | --- | --- | --- | --- | --- | --- | --- | --- | --- | --- | --- |
| CKX act. FIELD | 0.20 | 0.04 | -0.33 |  |  |  |  |  |  |  |  |
| *CKX1 field* |  | 0.04 | 0.28 | 0.08 | -0.13 | -0.27 | 0.22 | -0.16 | 0.28 | -0.24 | -0.06 |
| *CKX2.2.1 field* |  |  | 0.28 | **0.44*** | 0.36 | **0.57*** | **0.50*** | 0.08 | **0.52*** | -0.20 | 0.35 |
| *CKX2.2.2 field* |  |  |  | **0.68*** | 0.01 | 0.27 | **0.60*** | -0.13 | **0.74*** | -0.04 | 0.35 |
| *CKX3 field* |  |  |  |  | -0.20 | 0.12 | **0.52*** | -0.28 | **0.46*** | -0.17 | 0.26 |
| *CKX4 field* |  |  |  |  |  | **0.62*** | 0.23 | **0.48*** | 0.31 | 0.02 | 0.26 |
| *CKX5 field* |  |  |  |  |  |  | **0.43*** | 0.24 | 0.35 | 0.11 | 0.33 |
| *CKX8 field* |  |  |  |  |  |  |  | -0.13 | **0.69*** | -0.09 | 0.15 |
| *CKX9 field* |  |  |  |  |  |  |  |  | -0.17 | 0.09 | -0.10 |
| *CKX10 field* |  |  |  |  |  |  |  |  |  | -0.11 | 0.28 |
| *CKX11 field* |  |  |  |  |  |  |  |  |  |  | 0.18 |

C

| N34, seedling root | *CKX1 Root^1^* | *CKX3 Root^1^* | *CKX5 Root^1^* | *CKX8 Root^1^* | *CKX10 Root* | *CKX11 Root* | *NAC2 Root^1^* |
| --- | --- | --- | --- | --- | --- | --- | --- |
| CKX act. Root | 0.11 | **0.46*** | 0.05 | 0.20 | -0.13 | 0.19 | **0.46*** |
| *CKX1 Root^1^* |  | **0.50*** | **0.58*** | **0.55*** | 0.07 | -0.28 | **0.42*** |
| *CKX3 Root^1^* |  |  | **0.53*** | **0.58*** | 0.18 | 0.00 | **0.48*** |
| *CKX5 Root^1^* |  |  |  | **0.41*** | 0.05 | -0.27 | 0.35 |
| *CKX8 Root^1^* |  |  |  |  | 0.08 | 0.07 | 0.30 |
| *CKX10 Root* |  |  |  |  |  | -0.13 | -0.26 |
| *CKX11 Root* |  |  |  |  |  |  | -0.24 |

Table S3. Correlations among expression of *TaCKX* GFMs and *NAC2* in 7 DAP spikes from the growth chamber (A) and the field (B) on yield-related traits.  ^1^- non-parametric analysis; bold* – significant correlation at p ≤ 0.05.

A

| N34, 7 DAP GC | Tiller no. ^1^ | Spike no. ^1^ | Semi-empty spikes^1^ | Grain no. ^1^ | Grain yield ^1^ | Spike length ^1^ | TGW | CKX act. | *CKX1* | *CKX2.2.1* ^1^ | *CKX2.2.2* | *CKX3* ^1^ | *CKX4* | *CKX5* ^1^ | *CKX8* | *CKX9* | *CKX10* ^1^ | *CKX11* | *NAC2* |
| --- | --- | --- | --- | --- | --- | --- | --- | --- | --- | --- | --- | --- | --- | --- | --- | --- | --- | --- | --- |
| Plant height | 0.30 | 0.26 | 0.16 | **0.45*** | **0.59*** | **0.41*** | **0.46*** | **0.34*** | 0.15 | 0.17 | 0.18 | **-0.41*** | 0.01 | -0.09 | 0.14 | 0.00 | 0.03 | -0.05 | -0.22 |
| Tiller no. ^1^ |  | 0.02 | 0.02 | 0.24 | 0.30 | 0.20 | 0.10 | 0.07 | -0.21 | 0.12 | 0.28 | -0.04 | -0.19 | 0.15 | 0.04 | 0.37 | -0.29 | -0.21 | **-0.55*** |
| Spike no. ^1^ |  |  | **0.58*** | **0.77*** | **0.77*** | -0.02 | 0.12 | **0.38*** | -0.12 | 0.28 | 0.15 | 0.17 | 0.27 | 0.05 | 0.25 | 0.08 | -0.38 | 0.28 | 0.29 |
| Semi-empty spikes^1^ |  |  |  | 0.32 | 0.25 | -0.18 | 0.07 | **0.46*** | -0.09 | **0.57*** | 0.24 | 0.17 | 0.05 | **0.48*** | -0.26 | 0.12 | 0.16 | 0.10 | -0.05 |
| Grain no. ^1^ |  |  |  |  | **0.95*** | **0.36*** | 0.02 | 0.22 | -0.12 | 0.20 | 0.20 | -0.13 | 0.17 | 0.12 | 0.35 | 0.25 | **-0.47*** | 0.11 | 0.11 |
| Grain yield^1^ |  |  |  |  |  | **0.34*** | 0.28 | 0.28 | -0.06 | 0.12 | 0.22 | -0.15 | 0.17 | -0.07 | 0.30 | 0.15 | **-0.45*** | 0.06 | 0.07 |
| Spike length^1^ |  |  |  |  |  |  | -0.03 | -0.10 | -0.01 | -0.19 | -0.05 | **-0.42*** | 0.30 | -0.12 | 0.29 | -0.02 | 0.12 | -0.09 | -0.33 |
| TGW |  |  |  |  |  |  |  | **0.34*** | 0.17 | -0.02 | 0.12 | -0.13 | -0.14 | -0.31 | -0.16 | -0.18 | -0.08 | -0.12 | -0.18 |
| CKX act. |  |  |  |  |  |  |  |  | 0.16 | 0.14 | 0.04 | **-0.56*** | -0.17 | -0.11 | 0.08 | 0.24 | 0.13 | 0.22 | -0.19 |

B

| N34, 7 DAP field | Tiller no. ^1^ | Spike no. ^1^ | Semi-empty spikes^1^ | Grain no. | Grain yield ^1^ | Spike length ^1^ | TGW | CKX act. FIELD | *CKX1 field* ^1^ | *CKX2.2.1 field*^1^ | *CKX2.2.2 field*^1^ | *CKX3 field*^1^ | *CKX4 field*^1^ | *CKX5 field*^1^ | *CKX8 field*^1^ | *CKX9 field*^1^ | *CKX10 field*^1^ | *CKX11 field* | *NAC2 field*^1^ |
| --- | --- | --- | --- | --- | --- | --- | --- | --- | --- | --- | --- | --- | --- | --- | --- | --- | --- | --- | --- |
| Plant height | 0.30 | 0.26 | 0.16 | 0.29 | **0.59*** | **0.41*** | **0.46*** | -0.17 | 0.16 | 0.09 | -0.03 | 0.08 | 0.24 | 0.23 | 0.22 | -0.25 | 0.19 | -0.24 | -0.14 |
| Tiller no. ^1^ |  | 0.02 | 0.02 | 0.24 | 0.30 | 0.20 | 0.10 |  | -0.14 | **0.41*** | 0.28 | 0.29 | 0.23 | 0.30 | **0.39*** | 0.00 | **0.46*** | 0.14 | 0.04 |
| Spike no. ^1^ |  |  | **0.58*** | **0.77*** | **0.77*** | -0.02 | 0.12 | -0.54 | 0.18 | 0.14 | **0.34*** | 0.22 | -0.13 | 0.11 | **0.46*** | -0.26 | 0.26 | **-0.52*** | -0.04 |
| Semi-empty spikes^1^ |  |  |  | 0.32 | 0.25 | -0.18 | 0.07 | 0.02 | 0.16 | -0.07 | **0.33*** | -0.05 | 0.09 | 0.17 | 0.19 | 0.05 | 0.30 | 0.05 | 0.06 |
| Grain no. |  |  |  |  | **0.95*** | **0.36*** | -0.04 | -0.35 | 0.02 | 0.29 | 0.19 | 0.35 | -0.05 | 0.22 | **0.47*** | **-0.46*** | 0.38 | -0.27 | -0.10 |
| Grain yield^1^ |  |  |  |  |  | **0.34*** | 0.28 | -0.52 | 0.01 | 0.25 | 0.11 | 0.26 | 0.05 | 0.22 | **0.43*** | **-0.41*** | 0.29 | **-0.44*** | -0.08 |
| Spike length^1^ |  |  |  |  |  |  | -0.03 | -0.39 | -0.04 | 0.03 | -0.04 | 0.04 | 0.18 | 0.13 | **0.50*** | -0.35 | 0.29 | -0.14 | 0.03 |
| TGW |  |  |  |  |  |  |  | -0.11 | -0.08 | -0.23 | -0.18 | -0.20 | 0.37 | 0.11 | -0.09 | 0.06 | -0.07 | 0.06 | 0.30 |
| CKX act. FIELD |  |  |  |  |  |  |  |  | 0.20 | 0.04 | -0.33 |  |  |  |  |  |  |  |  |

Table S4. Correlations among expression of *TaCKX* GFMs and *NAC2* in seedling roots with seedling root weight and other yield-related traits. ^1^- non-parametric analysis; bold* – significant correlation at p ≤ 0.05.

| N34. seedling root | Tiller no. ^1^ | Spike no. ^1^ | Semi-empty spikes^1^ | Grain no. ^1^ | Grain yield^1^ | Spike length^1^ | TGW | CKX act. | Root weight | CKX act. Root | *CKX1 Root*^1^ | *CKX3 Root*^1^ | *CKX5 Root*^1^ | *CKX8 Root*^1^ | *CKX10 Root* | *CKX11 Root* | *NAC2 Root*^1^ |
| --- | --- | --- | --- | --- | --- | --- | --- | --- | --- | --- | --- | --- | --- | --- | --- | --- | --- |
| Plant height | 0.30 | 0.26 | 0.16 | **0.45*** | **0.59*** | **0.41*** | **0.46*** | **0.34*** | **0.49*** | 0.31 | 0.10 | 0.15 | 0.11 | 0.18 | -0.02 | -0.15 | 0.14 |
| Tiller no. ^1^ |  | 0.02 | 0.02 | 0.24 | 0.30 | 0.20 | 0.10 | 0.07 | -0.13 | 0.06 | -0.31 | **-0.39*** | **-0.41*** | -0.11 | -0.23 | 0.11 | -0.06 |
| Spike no. ^1^ |  |  | **0.58*** | **0.77*** | **0.77*** | -0.02 | 0.12 | **0.38*** | 0.12 | 0.17 | 0.06 | 0.22 | 0.01 | -0.08 | -0.34 | 0.24 | 0.16 |
| Semi-empty spikes^1^ |  |  |  | 0.32 | 0.25 | -0.18 | 0.07 | **0.46*** | 0.06 | **0.39*** | 0.13 | **0.45*** | -0.06 | -0.37 | -0.37 | -0.17 | 0.32 |
| Grain no. ^1^ |  |  |  |  | **0.95*** | **0.36*** | 0.02 | 0.22 | 0.16 | 0.06 | -0.08 | 0.05 | -0.12 | -0.12 | -0.16 | -0.01 | 0.04 |
| Grain yield^1^ |  |  |  |  |  | **0.34*** | 0.28 | 0.28 | 0.17 | 0.09 | -0.04 | 0.00 | -0.13 | -0.02 | -0.21 | -0.01 | 0.07 |
| Spike length^1^ |  |  |  |  |  |  | -0.03 | -0.10 | 0.03 | 0.10 | -0.25 | -0.17 | -0.05 | -0.02 | -0.05 | -0.33 | -0.05 |
| TGW |  |  |  |  |  |  |  | **0.34*** | -0.03 | 0.21 | 0.21 | 0.01 | -0.02 | **0.41*** | -0.06 | -0.17 | 0.24 |
| CKX act. |  |  |  |  |  |  |  |  | **0.43*** | **0.64*** | 0.30 | **0.43*** | 0.06 | **0.43*** | -0.12 | -0.06 | 0.21 |
| Root weight |  |  |  |  |  |  |  |  |  | 0.23 | 0.09 | **0.34*** | 0.06 | 0.25 | 0.20 | 0.01 | -0.11 |
| CKX act. Root |  |  |  |  |  |  |  |  |  |  | 0.11 | **0.46*** | 0.05 | 0.20 | -0.13 | 0.19 | **0.46*** |
| *CKX1 Root*^1^ |  |  |  |  |  |  |  |  |  |  |  | **0.50*** | **0.58*** | **0.55*** | 0.07 | -0.28 | **0.42*** |
| *CKX3 Root*^1^ |  |  |  |  |  |  |  |  |  |  |  |  | **0.53*** | **0.58*** | 0.18 | 0.00 | **0.48*** |
| *CKX5 Root*^1^ |  |  |  |  |  |  |  |  |  |  |  |  |  | **0.41*** | 0.05 | -0.27 | 0.35 |
| *CKX8 Root*^1^ |  |  |  |  |  |  |  |  |  |  |  |  |  |  | 0.08 | 0.07 | 0.30 |
| *CKX10 Root* |  |  |  |  |  |  |  |  |  |  |  |  |  |  |  | -0.13 | -0.26 |
| *CKX11 Root* |  |  |  |  |  |  |  |  |  |  |  |  |  |  |  |  | -0.24 |

Table S5. Correlations among expression of *TaCKX* GFMs and *NAC2* in 7 DAP spikes from the growth chamber (A) and the field (B) and expression in seedling root. ^1^- non-parametric analysis; bold* – significant correlation at p ≤ 0.05.

A

| N34 GC | *CKX1 Root*^1^ | *CKX3 Root*^1^ | *CKX5 Root*^1^ | *CKX8 Root*^1^ | *CKX10 Root* | *CKX11 Root* | *NAC2 Root*^1^ |
| --- | --- | --- | --- | --- | --- | --- | --- |
| CKX act. | 0.30 | **0.43*** | 0.06 | **0.43*** | -0.12 | -0.06 | 0.21 |
| *CKX1* | 0.15 | 0.14 | 0.18 | **0.46*** | 0.11 | 0.36 | -0.19 |
| *CKX2.2.1*^1^ | 0.16 | 0.21 | 0.23 | -0.12 | -0.11 | -0.24 | 0.02 |
| *CKX2.2.2* | **-0.33*** | 0.04 | -0.30 | 0.12 | -0.23 | 0.01 | -0.06 |
| *CKX3*^1^ | -0.25 | 0.06 | -0.10 | -0.14 | -0.15 | 0.15 | 0.23 |
| *CKX4* | -0.05 | 0.03 | 0.06 | -0.14 | -0.20 | 0.00 | 0.30 |
| *CKX5*^1^ | -0.13 | -0.01 | 0.13 | -0.29 | -0.16 | -0.25 | -0.15 |
| *CKX8* | **0.43*** | **0.44*** | 0.19 | 0.22 | 0.19 | -0.19 | 0.01 |
| *CKX9* | -0.05 | 0.10 | -0.12 | -0.09 | -0.33 | 0.02 | -0.13 |
| *CKX10*^1^ | 0.04 | 0.35 | 0.35 | 0.11 | -0.03 | -0.04 | 0.02 |
| *CKX11* | 0.21 | 0.19 | 0.38 | 0.27 | -0.19 | **0.39*** | -0.19 |
| *NAC2* | 0.10 | 0.22 | 0.19 | 0.02 | 0.25 | 0.15 | -0.13 |

B

| N34 Field | *CKX1 Root*^1^ | *CKX3 Root*^1^ | *CKX5 Root*^1^ | *CKX8 Root*^1^ | *CKX10 Root* | *CKX11 Root* | *NAC2 Root*^1^ |
| --- | --- | --- | --- | --- | --- | --- | --- |
| CKX act. FIELD | 0.43 | 0.41 |  |  |  |  | 0.59 |
| *CKX1 field*^1^ | **0.44*** | 0.32 | **0.59*** | 0.28 | -0.04 | -0.13 | 0.15 |
| *CKX2.2.1 field*^1^ | 0.11 | -0.16 | -0.29 | -0.09 | -0.26 | 0.08 | -0.25 |
| *CKX2.2.2 field*^1^ | -0.04 | -0.02 | -0.03 | 0.01 | -0.28 | 0.19 | -0.11 |
| *CKX3 field*^1^ | -0.01 | -0.24 | -0.01 | -0.05 | -0.08 | 0.18 | -0.34 |
| *CKX4 field* | 0.41 | 0.27 | -0.15 | 0.34 | -0.17 | -0.25 | 0.03 |
| *CKX5 field*^1^ | 0.17 | 0.03 | -0.29 | 0.16 | 0.03 | -0.24 | -0.03 |
| *CKX8 field*^1^ | 0.03 | -0.19 | 0.01 | -0.02 | **-0.49*** | -0.06 | 0.02 |
| *CKX9 field*^1^ | 0.26 | 0.14 | -0.01 | -0.07 | -0.06 | -0.07 | -0.04 |
| *CKX10 field*^1^ | 0.01 | -0.23 | -0.05 | 0.04 | -0.25 | -0.07 | -0.07 |
| *CKX11 field*^1^ | -0.07 | -0.08 | -0.22 | 0.00 | -0.04 | -0.06 | **0.53*** |
| *NAC2 field*^1^ | -0.05 | -0.20 | -0.27 | 0.30 | -0.02 | 0.08 | -0.22 |
